# United States Marine Hospital, San Francisco

**Published:** 1878-05

**Authors:** Edward J. Doering

**Affiliations:** Assistant Surgeon U. S. Marine Hospital Service; San Francisco


### UNITED STATES MARINE HOSPITAL, SAN FRANCISCO.

*Dermoid cyst.—Removal by knife.—Recovery.*

Erichsen, in his work on surgery, devotes a short paragraph to the subject of dermoid cysts, and states in substance, that these tumors occur occasionally; that they are congenital, and usually contain fœtal *débris*, as bone, hairs, etc.; that, finally, they are most frequently met with in the abdomen, about the face, within the skull, and in the lung, but never in connection with the extremities.

The following case may therefore be of interest by reason of the rare occurrence of this form of tumor.

The patient, Jno. Peters, æt. 27, a seaman, was admitted to the United States Marine Hospital last November, suffering from a mild attack of scurvy. An inspection of his body disclosed an immense tumor in the lumbar region, elastic and compressible, measuring 25 inches in one diameter, and 16 inches in the opposite. The patient stated that the tumor had existed since birth, when it was the size of an orange, and had gradually enlarged up to the present time. It had given him no inconvenience, except by its weight, for which reason he anxiously desired its removal. The following month, the patient having meanwhile recovered from his scurvy, Surgeon-in-charge C. N. Ellinwood, tapped the tumor to ascertain the nature of its fluid contents, withdrawing about three pints of a chocolate colored fluid, which on analysis proved, *not* to be cerebro-spinal, hence showing no connection with the spinal cord. The tumor was then removed at its base by the knife and galvanic cautery. The semi-solid contents remaining after the tapping, consisted of fatty matter and fœtal *débris*, principally hair, about 4 inches in length, growing from the interior of the tumor, the base of which was lined by a cuticular surface, studded with sebaceous glands, etc. The patient made a rapid recovery, and was discharged from the hospital, Feb. 16, 1878.

*Diffuse popliteal aneurism.—Amputation of leg.—Recovery.*

F. Grimes, æt. 32, colored, admitted to the hospital February 28, 1878, states that about two months ago, after more than usual exertion, he noticed a pulsating tumor in the left popliteal space. The tumor continued to grow in size, causing severe pains in the leg, but still the patient did not seek medical advice, but treated himself by using different liniments and patent medicines. Finally, about 10 days previous to his admittance, after sudden exertion, he felt something give way, and his leg became so much enlarged as to render him entirely helpless. On admission to the hospital, the left leg was found to be three times the size of the other, cold, brawny, and hard, the integument moreover showing strong signs of beginning mortification. In the popliteal space a tumor existed, which was solid and elastic, but without pulsation, confirming the diagnosis of diffuse popliteal aneurism.

The extravasation of blood evidently extending from the areolar tissue of the ham down to the foot, with no circulation in the limb by reason of the pressure of the effusion, and gangrene setting in, amputation of the leg remained as the only resource of saving the patient's life. Accordingly, Professor C. N. Ellinwood amputated the leg at the junction of the lower and middle third of the thigh. A dissection of the amputated leg showed complete obliteration of the popliteal vein, with disintegration of the deep fascia and muscles on the back of the leg, and complete rupture of the tendo achillis. The clots of blood within the aneurismal cavity, weighed five pounds. The patient, who is still under observation, made a splendid recovery, and will be discharged from the hospital this month.

Edward J. Doering, M. D.,
*Assistant Surgeon U. S. Marine Hospital Service.*
San Francisco, April, 1878.

